# Are Titania Photocatalysts and Titanium Implants Safe? Review on the Toxicity of Titanium Compounds

**DOI:** 10.3390/nano10102065

**Published:** 2020-10-19

**Authors:** Agata Markowska-Szczupak, Maya Endo-Kimura, Oliwia Paszkiewicz, Ewa Kowalska

**Affiliations:** 1 Department of Chemical and Process Engineering, West Pomeranian University of Technology in Szczecin, Al. Piastów 42, 71-065 Szczecin, Poland; po43902@zut.edu.pl; 2 Institute for Catalysis, Hokkaido University, N21, W10, Sapporo 001-0021, Japan; m_endo@cat.hokudai.ac.jp

**Keywords:** titanium alloys, titania, bioavailability, photocatalysis, environmental persistence, nanomaterials

## Abstract

Titanium and its compounds are broadly used in both industrial and domestic products, including jet engines, missiles, prostheses, implants, pigments, cosmetics, food, and photocatalysts for environmental purification and solar energy conversion. Although titanium/titania-containing materials are usually safe for human, animals and environment, increasing concerns on their negative impacts have been postulated. Accordingly, this review covers current knowledge on the toxicity of titania and titanium, in which the behaviour, bioavailability, mechanisms of action, and environmental impacts have been discussed in detail, considering both light and dark conditions. Consequently, the following conclusions have been drawn: (i) titania photocatalysts rarely cause health and environmental problems; (ii) despite the lack of proof, the possible carcinogenicity of titania powders to humans is considered by some authorities; (iii) titanium alloys, commonly applied as implant materials, possess a relatively low health risk; (iv) titania microparticles are less toxic than nanoparticles, independent of the means of exposure; (v) excessive accumulation of titanium in the environment cannot be ignored; (vi) titanium/titania-containing products should be clearly marked with health warning labels, especially for pregnant women and young children; (vi) a key knowledge gap is the lack of comprehensive data about the environmental content and the influence of titania/titanium on biodiversity and the ecological functioning of terrestrial and aquatic ecosystems.

## 1. Introduction

Titanium (Ti) is a transition metal with silver colour, high strength and low density. The most important property of titanium is its high chemical stability, i.e., titanium is even resistant to corrosion in sea water, chlorine and aqua regia. Naturally, titanium appears widely in the Earth’s crust and lithosphere, mainly in minerals, e.g., ilmenite, rutile and titanite. Metallic titanium is extracted from mineral ores mainly by Kroll and Hunter processes, i.e., by the reduction of titanium tetrachloride with magnesium and sodium, respectively. Titanium(IV) oxide (titania) is the most common titanium compound, widely used as a pigment and a photocatalyst. Other important titanium compounds are titanium chlorides, i.e., (i) titanium(IV) chloride (TiCl_4_), used as smoke screens and catalysts [1], and (ii) titanium(III) chloride (TiCl_3_), a catalyst for polypropylene synthesis [2].

**Figure 1:**
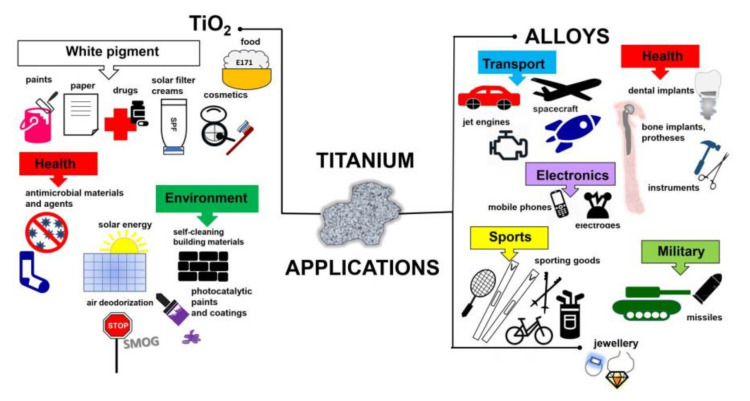
Schematic drawing showing miscellaneous applications of titanium-containing materials.

Titanium added to iron, aluminium, vanadium, molybdenum, tantalum and other metals forms lightweight and strong alloys, commonly used in aerospace (jet engines, spacecraft and missiles), metallurgy processes, dental/medical applications (prostheses, orthopedic implants, dental and endodontic instruments and files, dental implants), the car industry, agriculture, the military, sporting goods, mobile phones, jewellery and other applications [3,4,5], as shown in Figure 1. For example, Ouyang et al., (2019) have shown that Ti–Mg metal–metal composites facilitate osteoconduction and osseointegration (significantly higher around Ti-Mg than that around Ti implants) for orthopedic application [6]. Similarly, increased osseointegration has been observed on Ti_35_Zr_28_Nb alloy than that on pure titanium [7]. Moreover, Maharubin et al. [8] have proven that addition of silver (0.5–2.0 wt%) to titanium might limit post-surgery infection, one of the main causes of orthopedic implant failure. Additionally, titanium has been combined with other materials/compounds, such as organic compounds and polymers. For example, tannic acid-Ti/polysulfone membranes have been recommended for water remediation, especially for textile wastewater treatment, due to high hydrophilicity, excellent antifouling ability, powerful antimicrobial capability and good long-term stability [9]. Although, titanium and titania have already been used for various applications, their toxicity has not been addressed in detail, considering the direct and indirect impacts as well as the acute and chronic toxicity. Therefore, in this review, the toxicity of titanium/titania has been discussed thoroughly, based on recent literature reports and our own studies.

## 2. Toxicity of Titanium and Its Alloys

Titanium (Ti) has been widely used for building materials, parts of vehicles, and consumer goods (e.g., glass, camera and watches), cosmetics, drugs and dental/medical implants, due to its stability, low-density, mechanical strength, corrosion resistance and biocompatibility. Although some metals are essential biological elements, titanium has not played a biological role inside cells [10]. Moreover, it is widely known that titanium rarely causes allergic reactions in comparison to other metals. Osseointegration (binding between bone and titanium implant) without rejection was first reported by Branemark in 1983 [11]. Since this great discovery, titanium implant therapy has been developed intensively, e.g., for dental implantation, artificial joints and bones. It has been proposed that the formation of a passive film on the surface of titanium, due to the instantaneous binding of oxygen is the main reason for its lower allergic effect (lower ion release) than in the case of other metals [12,13,14].

The interest in Ti properties has been continuously growing, because of its use as an inert bio-implant material for medical and dental applications [15,16,17,18,19]. The evidence of titanium toxicity has not been reported for many years, and thus titanium has been considered as an inert material with high biocompatibility. However, in rare cases, allergic symptoms, caused by titanium (alloy) implants, have been suggested, e.g., irritation, inflammation, erythema, lichenoid reactions and so on [20,21,22,23]. Additionally, it has been reported that titanium could corrode under some conditions, e.g., low pH, in the presence of fluorine, or in the contact with other metals [14,20,23]. Interestingly, Hanawa (2004) has suggested that a release of metal ions does not necessarily damage the human body, however, their binding to biomolecules could be toxic [13]. It is known that Ti ions exhibit high activity, reacting with hydroxyl radicals and anions immediately, and thus the trace amount of Ti ions might react with biomolecules, inducing body injury [13]. Indeed, the titanium release from hip-replacement components has resulted in titanium accumulation in serum and hair of patients with titanium alloy implants [24], probably because of the long-distance “travelled” by titanium [25]. Additionally, it has been suggested that the released titanium ions show high affinity for serum transferrin, binding the protein through metal binding sites [26].

There are many indications that titanium might cause some problems, e.g., “yellow nail syndrome” (YNS), allergic and autoimmune reactions. In view of this, it is possible and even necessary to discuss the toxicity and the allergy caused by titanium and its alloys [19]. Although numerous papers on titanium have been published, the chronic or sub-chronic effects on organs and various types of tissue, the dose-response correlations, and models of action have not been fully elucidated. Due to widespread use of titanium implants in prosthodontics and orthopedics, the most valuable data could be found in respective medical papers. It has been well known that titanium implants are in direct contact with body fluids (saliva) that contain various inorganic and organic compounds. In addition, the implant surfaces can be inhabited by bacteria, which might initiate the corrosion [27]. Although, titanium alloys are generally considered as passive under normal physiological conditions, some exceptions, such as oxide layer disruption or oral implant corrosion, have been reported [15,17]. For example, low pH, high concentration of fluoride (dental implants) and the presence of oxidizing agents are considered as the main factors inducing corrosion [28,29,30]. Moreover, it has been found that the toxicity of titanium alloys depends on the alloy composition [16,31,32,33,34,35], and thus the careful selection of the material should be performed [18]. The first-generation titanium alloys, which contain Cr, Ni, Be and Co, are very toxic, whereas those with Al and V exhibit little toxicity and slight allergic effects. On the other hand, new titania alloys containing Nb show favourable osteoconductive and osteoinductive properties due to the formation of an apatite layer on their surfaces, when exposed to an acidic environment [36]. Moreover, other cations (e.g., Ag, Cu, Zn and Ce) might present additional therapeutic effects, e.g., angiogenesis that is essential for cicatrize process, and antimicrobial properties [18,37]. According to Ikarashi et al. [38], titanium–zirconium (Ti–Zr) alloy-implants exhibit the best biocompatibility, improved properties (in respect to pure Ti) and a low level of fretting corrosion. Nowadays, toxicological effects, related to antibacterial properties of noble metals’ ions, such as Ag^+^ and Au^+^, which might be released by titanium alloys, have been a growing matter of concern [39]. Similarly, nanostructures/compounds containing antibiotics with the antibacterial, anti-infective and anti-inflammatory properties, have been under consideration. Although antibiotics are used to control invading organisms (mainly bacteria and protozoa) on the surface of implants, very often they cause some problems, including cell toxicity, allergic response, impairment of osteogenic activity and antibiotic-induced adverse reactions, e.g., superinfections and hypersensitivity [40,41,42].

The risk assessment of titanium and titanium alloys requires the quantification of unintended effects associated with a release of particular components. Obviously, this is not an easy task since very often contradictory data have been provided. For example, Rae [43] has postulated that pure titanium and titanium alloy (Ti-6Al-4V) do not affect human fibroblast cultures because of the relatively-low solubility of Ti ions [43]. By contrast, the corrosion products of titanium implants have been identified in serum and bone marrow, then being transported through the bloodstream and/or lymph to hair, lungs, spleen, liver and kidneys [44,45,46]. Accordingly, titanium has been detected in inner organs, including lungs, kidneys and liver, five months after a dental implant placement because of the translocation mechanisms [47]. Therefore, an estimation of changes in the content of Ti in the blood exposed to bone and dental implants, has been proposed as one of the toxicity indicators. Unfortunately, the changes of titanium content in the blood do not correlate with the implant-bone contact area, implants’ number and gender [48,49].

Other symptoms of implants’ corrosion include periprosthetic osteolysis, implant loosening and increased expression of proinflammatory mediators such as interleukins, prostaglandins, monocyte chemotactic proteins and macrophage colony stimulating factors [50,51,52,53]. Moreover, the particles of dental implants have been considered as initiators of destructive inflammatory processes, affecting tissues that surround dental implants—peri-implantitis [54,55,56]. For example, 100–300 ppm of titanium has been detected in trigger tissues [57]. The contact allergy to titanium might lead to pain, eczema, atopic dermatitis, swelling, erythema, urticaria and weakening of implants [15,57,58,59,60]. However, a difficulty in assessment of Ti allergy, because of uncertainty of the detection methods, seems to be the main problem [16]. In order to prevent implant failure, attention should be paid to a patient’s medical history to indicate the multiple allergies, e.g., to metals and jewellery [61].

A few studies indicate a possible connection between titanium and YNS [62,63,64]. The YNS or lymphedema associated with yellow nails is an uncommon and rare medical syndrome [63], characterized by slow nails’ growth, their yellow discoloration, lymphedema and tract involvement. In 2011, Berglund found a correlation between the titanium content in nail clippings and the yellowness and/or thickness of the nails. An excessive exposure to titanium from orthopedic implants along with ingestion of some drugs and foods (e.g., chewing gums, candies, chocolates) might be given as a probable cause of YNS. It seems extremely likely that the synergistic effect of chronic subthreshold is relevant. Moreover, fluoride-containing toothpastes and fluoride gels used for oral hygiene might exacerbate YSN. Berglund [62] has shown that titanium implants are a source of titanium ions, which are released from implants because of the galvanic action of dental gold and amalgam or oxidative reaction with fluorides [62]. Interestingly, the symptoms disappeared after stopping the galvanic reactions of titanium with other metals, and thus an exposure to titanium. Moreover, YNS might be dependent on underlying genetic and immunological disposition [63,64]. The experiments on animals have confirmed the titanium release from dental and orthopedic implants [27,56,65,66]. Furthermore, it has been found that the surface roughness of a metal insert is the most important factor of titanium release from the implant surface, i.e., the rougher the surface is, the higher is the coefficient of friction, and thus titanium release. In contrast, total area and diameter of implants are less important [66].

Titanium plasma-sprayed (TPS) implants should be considered as a special case, due to gradual and passive dissolution of their surface, which results in a decrease in the size of titanium particles with an increase of the distance from the implant surface. For example, titanium particles, released from TPS implants, have been detected at the average distance of 200–250 µm (till 500 µm) from the implants’ surface [67]. The analysis of histological sections has shown the presence of titanium at the distance from 0.4 mm up to 4.0 mm [27]. Generally, the Ti particles’ size, found in animal and human tissues, ranges from 10 nm to 54 µm [56]. It has been reported that Ti particles have a cytotoxicity effect through reduction of bone marrow stem cells (BMSCs) viability and proliferation, increase of p53 protein level, disruption of cell homeostasis and induction of DNA damage [68]. For example, Gomes et al. [69] showed (through an in vitro study) that Ti-6Al-4V alloy, widely used in medical and odonatological implants, presents a cytotoxic effect, i.e., the DNA damage (breaking of DNA strands) and mitotic spindle, leading to loss of whole chromosomes during cell division. However, the model of action is still unknown.

Two mechanisms of metal ions’ interactions with DNA have been considered: (1) direct and (2) indirect actions [70,71,72,73]. (1) Titanium as a transition metal (d-block metal) has incomplete d-orbital, and thus can bind directly to the DNA bases (N7 of purine or N3 of pyrimidine atom at G-C sites). On the other hand, (2) titanium has low-energy d orbitals, which suggests that indirect mechanism is more probable, i.e., based on increased formation of reactive oxygen species (ROS), and formation of hydrogen bonds between the coordinated ligands and negatively charged phosphate groups in DNA structure [38,70].

**Figure 2:**
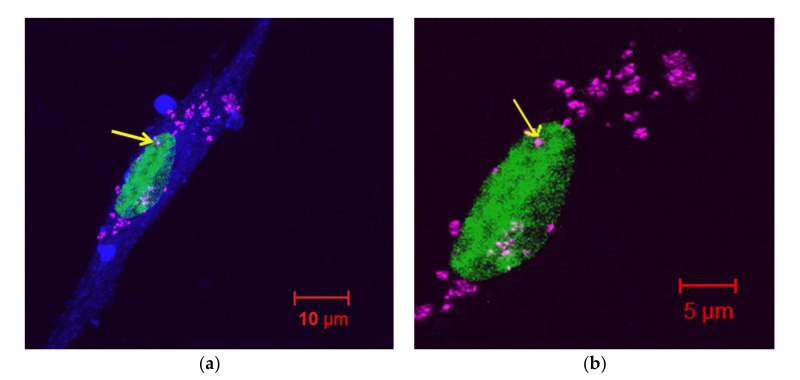
Laser scanning confocal microscopy image of: (**a**) a periodontal ligament human telomerase reverse transcriptase (PDL-hTERT) cell after exposure to titanium nanoparticles (Ti-NPs, 28 μg/ml) for 24 h (z-stack slices merged into one image). Ti-NPs: pink, cell plasma membrane: blue, nucleus: green, yellow arrow: Ti-NPs in the nucleus; (**b**) the nucleus of the same cell in (a). Ti-NPs: pink, nucleus: green, yellow arrow: Ti-NPs in the nucleus. Adapted from reference [73] with permission from Elsevier, 2015.

Considering the methods of toxicity evaluation, both Ti (alloy) particles and Ti ions have been investigated in vitro, ex vivo and in vivo, i.e., on DNA/RNA, protein, lipids, cells and animals. Accordingly, the cellular incorporation of titanium has been well studied, e.g., the cellular uptake efficiency is higher for titanium nanoparticles (NPs < 100 nm) than titanium microparticles (<44 μm). Moreover, only NPs have been observed in the nucleus, as shown in Figure 2, with 352 times higher cytotoxicity than microparticles [73]. However, it should be mentioned that large titanium particles could be incorporated into cells by phagocytosis [74]. Evans has evaluated the effect of titanium (mean size of 49 μm), ground titanium (14 μm) and titanium alloy (Ti90/Al6/V4, 8.9 μm) on the cell viability using two experimental conditions, i.e., (1) in the direct contact with cells, and (2) separated from them [75]. Although large titanium does not cause a decrease in the cell number under both conditions, small titanium significantly reduces the number of cells when they are in contact with titanium. Moreover, titanium alloy causes a higher reduction of cell number than ground titanium when in contact with cells. Accordingly, it has been proposed that small particles (5–10 μm) could induce cell damage by direct contact. The size-dependent cytotoxic effect of titanium particles/ions on neutrophils has also been shown, i.e., the fine titanium particles (1–3 μm) are incorporated into cells by phagocytosis causing the cytotoxicity [74,76], whereas Ti ions stimulate neutrophils and increase the quantity of released superoxide anions [74]. Moreover, it has been shown that the intraperitoneal injection of titania suspension induces the uptake of titanium by the blood cells (macrophages and phagocytic mononuclear cells) and its further dissemination to organs, such as liver, spleen and lungs via cells [44].

The cellular response to titanium particles/ions has been investigated mainly for oral mucosa cells. For example, it has been found that the exposure of mouse osteoblast-like MC3T3-E1 cells to Ti ions inhibits cell proliferation (in dependence on the concentration and time), and induces nuclear expression of Yes-associated protein YAP (a key transcription co-activator, the activity of which is inhibited by the Hippo signaling pathway) in osteoblasts, resulting in down regulation of osteogenic differentiation of MC3T3-E1 cells [77]. According to the in vivo study on detection of lactate dehydrogenase (LDH), interleukin (IL) and activated nuclear factor-kappa B (NF-κB), inflammatory reaction (high content of IL-6) and activated NF-κB have been detected around a titanium implant [78]. Moreover, it has been proposed that TNF-α, IL-1β, and IL-6 might induce osteoclastogenesis and inhibit osteoblastogenesis through the RANK–RANKL (receptor activator of nuclear factor kappa-Β–receptor activator of nuclear factor kappa-Β ligand) pathway [79]. Therefore, it has been concluded that titanium might induce inflammation. Moreover, it has been proposed that cells’ exposure to titanium might also influence the content of proteins and lipids. Indeed, titanium has caused a decrease in total protein content and some types of lipids, e.g., cholesterol ester and phosphatidylethanolamine, inducing the potential damage of tissues [80].

López-Jornet et al. [81] evaluated the DNA damage by dental implants in gingival cells, collected from patients with implants. The concentration of titanium (Ti^47^) in these cells was significantly higher than that in control cells (from patients without implants). The frequencies of micronuclei and binucleated cells, and nuclear buds in the “implant” group, have been higher than those in the control group, but without statistically significant differences. Moreover, during the study on the effect of Ti ions on osteoblast, Liao et al. [82] have revealed that the equal or higher concentration of Ti ions than 10 ppm inhibits cell proliferation. Additionally, it has been found that Ti ions: (i) reduce the expression of osteonectin and osteopontin mRNAs, (ii) delay the development of alkaline phosphatase mRNA expression, and (iii) decrease the enzyme activity.

**Figure 3:**
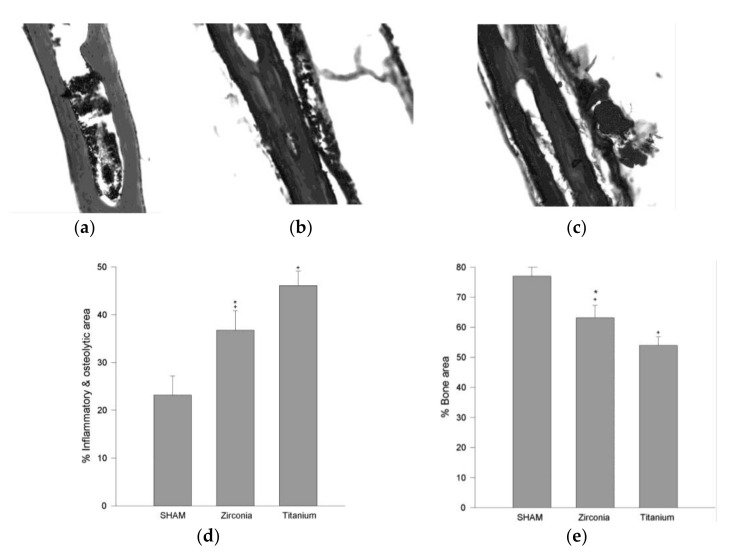
(**a**–**c**) Histological microscopic images of murine calvaria stained with hematoxylin and eosin (magnification: ×200): (**a**) In sections obtained from PBS-treated animals (sham group); (**b**) In sections obtained from zirconia treated animals; (**c**) In sections obtained from titanium treated animals; The quantitative evaluation of tissue reactions, expressed as percentage of (**d**) osteolytic and (**e**) bone area; + indicates significant difference to sham group and * indicates significant difference to titanium treated group; One way analysis of variance (ANOVA) and Tukey’s test, *p* < 0.05. Adapted from [79] with permission from Elsevier, 2014.

It should be remembered that the toxic symptoms due to titanium are not only allergic reactions, but also disorders in a whole body. Fretwurst et al., (2016) have proposed that a release of Ti ions could participate in peri-implant bone loss [83]. Additionally, acidic conditions in an oral cavity might increase the corrosion of titanium [84]. Moreover, the induction of osteoclastogenesis and the inhibition of osteoblastogenesis can lead to bone resorption around joint replacements [79]. As shown in Figure 3, mice treated with zirconium and titanium have expressed an inflammatory reaction and the reduction of bone surfaces in comparison to a sham group (PBS treated).

Despite the abundant content of titanium in the Earth’s crust, water contamination by abnormal content of titanium might also affect human health. Titanium has been found in river water, and thus accumulated in aquatic insects [85]. Therefore, its possible impacts on the food chain and the agricultural damage must be considered. Moreover, a statistical study in Mexico has suggested that titanium in the blood, ingested by insufficiently treated water, might be related to low haemoglobin content, and thus anaemia in children [86].

The effect of titanium on bacteria cells has also been investigated, but contradictory results have been reported, i.e., (i) no significant bactericidal effect on oral bacterial species [87,88], and (ii) bactericidal activity of titanium [89]. Recently, Stolzoff et al. [84] have revealed the effect of surface topography of titanium on bacteria. It has been found that a high density of uniformly sized nanofeatures prevents bacterial adhesion and proliferation [90]. Considering that bacteria might cause implant failure, therefore, the development of bacteria-resistant titania implants would be highly valuable for patients.

Summarizing, titanium is one of the safest metals, as it has been widely used for clinical implants. However, it is necessary to consider and evaluate carefully all possible negative impacts on human body. Moreover, clinicians should pay attention when titanium-based implants are installed in patients.

## 3. Toxicity of Titanium(IV) Oxide

**Figure 4:**
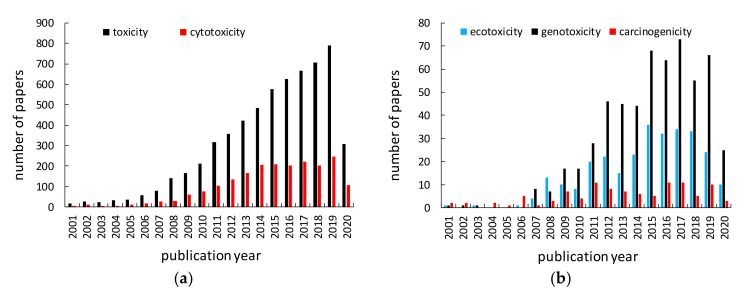
Number of papers published annually on titanium toxicity searched in Web of Science (May 25, 2020) using: (**a**) “titanium dioxide toxicity” or “TiO_2_ toxicity” or “titania toxicity” (black) and “titanium dioxide cytotoxicity” or “TiO_2_ cytotoxicity” or “titania cytotoxicity” (red); (**b**) “titanium dioxide ecotoxicity” or “TiO_2_ ecotoxicity” or “titania ecotoxicity” (blue), “titanium dioxide genotoxicity” or “TiO_2_ genotoxicity” or “titania genotoxicity” (black) and “titanium dioxide carcinogenicity” or “TiO_2_ carcinogenicity” or “titania carcinogenicity” (red).

Titania (titanium(IV) oxide, titanium dioxide, TiO_2_) is the most widely used titanium compound, and thus its toxicity should be carefully examined. Similar to titanium, titania has been reported as inert, and thus safe for humans and the environment for many years. Non-toxicity of titania has been listed as one of the main advantages of titania photocatalysts among high activity, chemical and thermal stability, broad availability and low costs. However, considering the nanoparticulate nature of titania photocatalysts, the nanosize-governed toxicity of titania has been postulated [91,92]. Accordingly, various studies on titania toxicity have been performed, as shown in Figure 4. More than 6000 papers have been published on “titania (titanium dioxide, TiO_2_) toxicity” (searched in Web of Science), and about 60% of them (3674 results) in the last five years. Accordingly, an evaluation of cytotoxic, ecotoxic, genotoxic and carcinogenic potential of TiO_2_ has been represented in large number of scientific papers (Figure 4). However, it should be pointed out that the possible toxicity of titania has been intensively studied only in the last few years, and some potential effects are still unknown, e.g., carcinogenicity (only ca. 10 papers/year), which might suggest the low toxic effect of titania. Accordingly, the possible hazardous impact of TiO_2_ has been reviewed and discussed in the following sections, including carcinogenicity.

### 3.1. Can Inhalation of Titania Cause Cancer?

Pure titania pigment is a fine white powder, which looks like icing sugar. Typical TiO_2_ is characterized by a primary particle size between 0.2 and 0.5 µm (nanostructured titania has nanometre size, i.e., about 5–40 nm). However, a dusty form of titania could become airborne, and thus be easily inhaled. For this reason, titania (similar to other insoluble dusts, e.g., carbon, diesel black exhaust) has been considered as a potential health hazard [93].

*A* carcinogen is any substance, radiation or radionuclide, that promotes the formation of cancer (Carcinogenesis, oncogenesis or tumorigenesis). In the majority of cases, carcinogens do not cause acute toxicity (are not immediately toxic). Although titania has been considered as non-toxic, titania NPs of sizes up to 20 nm have been classified as a possible carcinogen to humans (Group 2B carcinogen) by the World Health Organization’s International Agency for Research on Cancer (IARC) since 2006. Accordingly, in 2015, the French Agency for Food, Environmental and Occupational Health and Safety (ANSES) requested recognition of TiO_2_ as a carcinogen by inhalation to the Committee for Risk Assessment (RAC), which has prepared the opinions for the European Chemicals Agency (ECHA). The first time that the issue was discussed by RAC was at the meeting in March 2017. However, the ECHA committee stated (Helsinki, 9 June 2017) that the potential of titania as a carcinogen was less restrictive than presumed carcinogens (Group 1B carcinogen), and more research and requirements in classification, labelling and packaging (CLP) have been requested. The important aspect of this dispute was that the Titanium Dioxide Manufacturers Association (TDMA) pointed out that only rodent studies indicated the carcinogenic potential of TiO_2_ [94,95]. Finally, during the meeting of the CARACAL group (Competent Authorities Meeting for REACH and CLP regulations) on 18 September 2019, TiO_2_ was classified as a category 2 carcinogen, due to its inhalation hazard: “The classification is reported to apply to liquids as well as powders containing 1% or more of titanium dioxide in the form of or incorporated in particles with aerodynamic diameter ≤10 µm”. Accordingly, the appropriate act was submitted to the European Parliament and Council [96].

Similarly, the National Institute for Occupational Safety and Health (NIOSH), the United States federal agency responsible for the prevention of work-related injury and illness, has recommended the exposure limits of 2.4 mg/m^3^ for fine TiO_2_ and 0.3 mg/m^3^ for ultrafine (including NPs) TiO_2_ as the time-weighted average (TWA) concentrations (TWA is a method of calculating a worker’s daily exposure to a hazardous substance or agent, averaged to an 8-hour workday, taking into account the average levels of the substance or agent and the time spent in the area. A time-weighted average is equal to the sum of the portion of each time period (as a decimal) multiplied by the levels of the substance or agent during the time period divided by the hours in the workday) for up to 10 h per day during a 40-h work week. This means that over a working lifetime the risk of exposure is less than one person in 1000. The Occupational Safety and Health Administration (OSHA) has recommended an exposure limit (PEL) at 15 mg/m^3^ for TiO_2_ as a total dust and 5 mg/m^3^ as a respirable dust. Much lower limits have been applied in Germany, where the MAK (Maximum Concentration Values in the Workplace) values are 4 and 1.5 mg/m^3^, respectively. Since any exposure might involve some degree of risk, the general recommendations must result in the exposure reduction to the lowest achievable levels. As pointed by Hex et al. [93], the average concentration of respirable TiO_2_ dust depends on many factors, and varies in time (calendar year) and place (TiO_2_ factory). The median cumulative exposure of workers has been estimated at 1.98 mg/m^3^ × years, which is much less than the recommended exposure limits [93].

To better understand how titania affects human health, animal models, in particular rats or other rodents (mice and hamsters), have been used as exemplary for commercial titania, as presented by Relier et al. [97]. Accordingly, obtained data allow us to compare various conditions of TiO_2_ exposure and multiple exposure routes, and help to assess the model of action, even when the animal model of the organ injury does not correspond to the human organ injury or identification of conserved gene expression across organisms.

The importance of titania routes of exposure has been considered as follows: inhalation > oral exposure > dermal exposure (skin). Gas exchange, which involves an inhalation of oxygen and a release of carbon dioxide, is conducted by a respiratory system, which is a primary target structure. The sub-chronic and chronic effects have revealed that titania can be deposited in the lungs, and thus can be responsible for their injury, leading even to cancer development [98].

According to the National Library of Medicine (NLM)—Toxnet, there is only limited adequate evidence for the carcinogenicity of titania to animals’ respiratory systems [99]. Some human cases of lung injury, caused by the exposure to titania in various forms, are listed chronologically in Table 1. The mortality rate for these 13 cases (Table 1) is unknown.

The vast majority (85%) of cases of lung cancer are caused by smoking or exposure to the smoke (secondhand smokers), whereas about 10–15% cases are due to a combination of genetic factors, confirmed by family history of lung cancer, or exposure to radon, arsenic, chromium and nickel or other forms of air pollutants. In practice, it is difficult to obtain a clear correlation between the mortality and the occupational exposure to titania, because in several cases, the smoking habits of study subjects have not been reported. The statistically valid epidemiologic studies of the mortality caused by titania were conducted by Chen and Fayerweather [104], Boffetta et al. [105] and Fryzek et al. [106]. In the first case, 1576 employees exposed to TiO_2_ were observed from 1956 to 1985 for cancer and chronic respiratory disease incidences, and from 1935 to 1983 for mortality. No cases of pulmonary fibrosis were found among TiO_2_-exposed employees. In the second case, 15,017 workers (14,331 men) employed in 11 factories producing TiO_2_ in Europe were examined. It was assumed that among men, the mortality from lung cancer did not increase with an increase in an employment duration or an estimated cumulative exposure to TiO_2_ dust. In the last study, 4241 workers, who were employed for at least 6 months in four TiO_2_ plants in the United States, were analyzed. It was found that employees with a higher exposure to titania had similar mortality rates to those with lower exposure. Therefore, in all these studies the same conclusion has been drawn, i.e., titania dust does not present a significant carcinogenic effect on human lungs.

**Figure 5:**
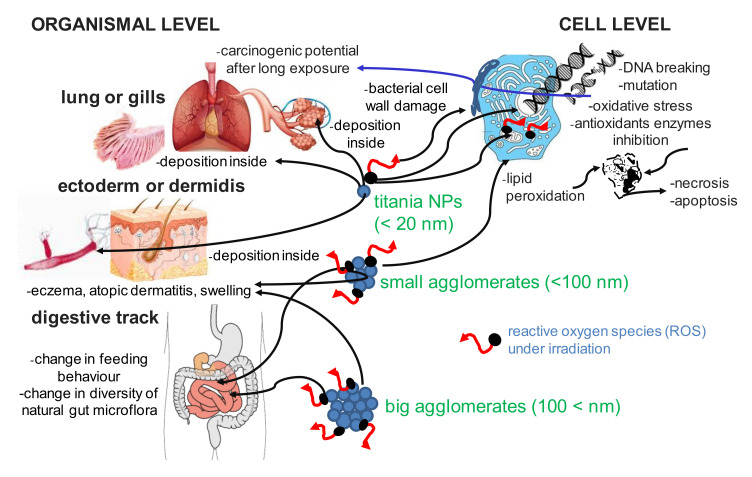
Comparison of titanium dioxide impact on cell and organismal level.

The influence of titania properties, including the size, the surface area, the surface chemistry and charges, the crystallinity, the shape, the solubility and the agglomeration/aggregation state, on lung injury has been summarized in the review paper by Wang and Fan [107]. The most important conclusions from this work are: (i) titania NPs with the smaller sizes than 20 nm and small titania agglomerates (<100 nm) present the higher carcinogenic potential than fine TiO_2_ particles (ca. 200–250 nm), due to the induction of oxidative stress and DNA single and double-strand breaks; (ii) morphologically-ordered titania, including zero-, one-, two- and three-dimensional (0-D, 1-D and 3-D) assemblies as nanospheres, nanorods, nanotubes and nanobelts present varied effects in their deposition in the lungs (time- and shape-dependent); (iii) both anatase and rutile (two main polymorphs of titania) show the induction of inflammatory responses, cytotoxicity and ROS formation that lead to cell necrosis or initiate apoptosis; (iv) the ability of anatase to decrease the lung cell viability is slightly larger than that of rutile, (v) the surface modification (or surface coatings with inorganic or organic compounds) is an important factor influencing the toxicity of titania in the respiratory system; (vi) the main factors inducing the cytotoxicity (or oxidative stress induction) and genotoxicity are ROS, generated when TiO_2_ is light-activated; (vii) the crystalline forms of titania, the size, the specific surface area, the number of defect sites, etc. influence ROS formation. The toxicity of titania in dependent on the size of titania particles and the place of impact is exemplary as shown in Figure 5. Considering the surface charge of particles, Fröhlich [108] has postulated that there is no rigid rule of charge-dependent particle uptake, but it seems that cationic surfaces of NPs (not only TiO_2_) result in larger cytotoxicity.

In summary, it should be pointed out that there is very little evidence that might link the increased mortality from lung cancer with the increased exposure to TiO_2_ dust. Additionally, a cumulative effect from airborne pollutants, e.g., nitrogen oxides, sulphur oxides, ozone, smoke, might cause (together or separately) severe sickness and premature death. For these reasons, although caution is advised, titania dust cannot be unambiguously defined as a human lung carcinogen.

### 3.2. Dermal and Oral Exposure to Titania

One hundred and fifty items of daily cosmetic products containing titania, such as toothpaste, sunscreens, food and beverage colorants, drugs, vaccines and nutritional supplements with long-term contact with human organs (e.g., skin, digestive track), have been examined by Shi et al. [109]. It should be pointed out that direct contact is also possible during manufacturing of titania (e.g., bagging, handling, labelling, sampling, overlaying). Accordingly, many studies involving skin lines have been conducted [110,111,112,113], and in vivo studies provide accurate and reliable data, due to the possibility to follow the course of active penetration during long-term exposure. In contrast, in vitro studies have a few limitations, e.g., (i) only passive distribution through skin layers can be verified; (ii) they are only limited to one cell type (e.g., keratocytes); and (iii) only short-term experiments can be conducted. Based on the results obtained from both types of experiments, it is thought that titania particles could not access intact skin through intercellular channels of stratum corneum, sweat glands and hair follicles [114,115]. Moreover, only the accumulation of titania NPs on the skin surface has been observed, which is caused by titania tendency to form aggregates, and additionally confirming the great stability of titania [110,115]. However, it could be basically imagined that scratching, small wounds, bites, burns and other slight damage might break the skin and facilitate titania penetration. However, in vitro and in vivo results, obtained by Xie et al. [113], have shown that 20-nm titania NPs could not penetrate skin even when its layers are damaged, Senzui et al. [111] have found that the concentration of titanium in skin increases when it has been applied on hair-removed skin. Moreover, TiO_2_ from cosmetics could pass through hair follicles and pores (greater than 1 mm) but has not been detected in dermis and viable epidermis layers [110,111]. Therefore, it has been proposed that the form of titania is crucial for the penetration rate. According to Bennat and Muller-Goymann [110], titania NPs applied as an oil emulsion become deeper inside the skin than that in the form of an aqueous colloid.

From the late 1990s, TiO_2_ and ZnO NPs from sunscreens have been postulated to be involved in the generation of free radicals’ (including singlet oxygen) in skin cells [98,116,117,118,119], and thus resulting in possible protein dysfunction, mutations, direct DNA damage, and the further tumorigenesis and the cancer development. This is a kind of perversity since sunscreens are used for skin protection to prevent sunburn and to reduce skin cancers. However, it should be noticed that an evaluation of ROS influence on direct tumour formation is rather difficult, due to their short lifetime [120]. Interestingly, titania has been proposed as an efficient agent for possible local treatment of cancer, e.g., an increase in cells’ mortality and significant morphological alterations in living cells for MCF7 (breast adenocarcinoma) have been observed with an increase in titania dose (from 10 to 50 mg/μL) and under ultraviolet (UV-A) irradiation (as compared with experiments in the dark) [121].

**Figure 6:**
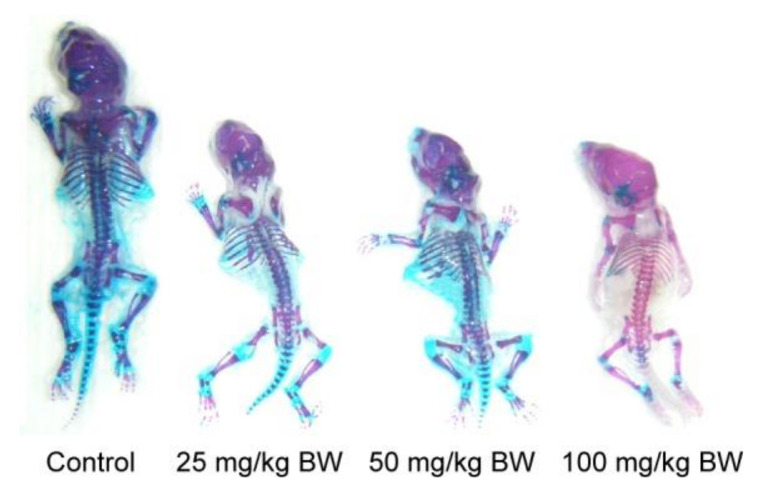
Stereo microscopic images after treatment by Menegola’s method of mice embryos that were maternally exposed to nano-TiO_2_ at gestational day 18. Blue, cartilage. Purplish red, ossification. Red, incomplete ossification. Adapted from reference [135] with permission from Dove Medical Press, 2017.

The average dietary exposure to TiO_2_ (E 171) from its use as a food additive is between 0.4 and 10.4 mg of TiO_2_ per kg of body weight per day throughout life. Obviously, children are the most exposed group of studied individuals [122]. Growing concerns, related to acute and sub-chronic toxicity in rodents by their exposure to TiO_2_ through food, have already been noted [123,124,125,126,127,128]. Fortunately, no acute toxicity has been reported. For a sub-chronic effect, 28- and 90-day tests have been performed for male and female rats, dosed with rutile-type pigment of particles’ size from 73 nm to 145 nm, for no-adverse-effect level (NOAEL) from 1000 mg/kg bw/day to 24,000 mg/kg bw/day. Generally, negligible toxicological effects have been observed. However, LD_50_ (rat) value has been given for oral exposure to titania, reaching >5000 mg/kg bw [129]. Indeed, it has been found that an oral exposure of nano-TiO_2_ to pregnant mice clearly induces the dysplasia of skeleton and suppresses the development of mice embryos, as shown in Figure 6. Interestingly, the transport to other tissues outside the digestive system (e.g., lung tissues, spleen, kidney, brain and lymph) has also been confirmed [129]. Geraets et al. [124] have revealed another warning, i.e., slow elimination and potential accumulation of titania in tissues. The significant changes in body parameters (e.g., decrease in body weight gain) and the factors affecting fertility (e.g., an increase in sperm abnormalities, a decrease in a number and size of the epithelial lining of the tubuloalveolar gland and hyperplastic glandular epithelium of the seminal vesicle, and a decrease of sperm cell concentration and serum testosterone level) have also been reported after continuous feeding of the male albino rats with food containing 1–2% titania for 65 days [130]. An increase in the levels of dopamine and norepinephrine in the brain cerebral cortex appears to be the the most alarming symptom of possible neurotoxicity [125]. The results obtained by Mohammadipour et al. [131] have proven this hypothesis to a certain extent, i.e., rat offspring being forced to consume 100 mg/kg titania for 21 days impaired memory and learning abilities. Moreover, Bideskan et al. [132] have found that exposure to TiO_2_-NPs during rat pregnancy and lactation periods induces apoptosis and decreases neurogenesis in hippocampus. In this regard, it has been proposed that pregnant and lactating women should avoid food additives containing titania, such as E171 [133].

Additionally, it should be pointed out that these aspects should be considered when titania is proposed as an antimicrobial agent (bare and modified titania, e.g., with NPs of noble metals) for applications connected with food protection against spoilage, e.g., for food storage containers [134].

The physicochemical characteristics of titania NPs, which might influence gastric toxicity, have been studied by many teams [136,137,138,139]. For example, it has been found that the properties of titania influence its entry pathway and further body penetration. The main titania absorption in the small and large intestines takes place through: (i) villi and microvilli of the epithelium and (ii) the lumen of the intestinal tract via aggregation of intestinal lymphatic tissue and the normal intestinal enterocytes. Moreover, the impact of a rutile form on gut microbiota is more pronounced than that of an anatase form. However, the reason for this phenomenon is unclear. It has also been reported that toxicity, induced by titania, might be attributed to mainly high dosing rather than the particle size and distribution [124]. However, Wang has found that for rats the 80 nm NPs of titania are more harmful than 25 nm ones [107].

An oral exposure to titania might induce an oxidative stress and alternation of enzymes activity. The changes in enzymes activity have been recognized for lactate dehydrogenase (LDH), alanine aminotransferase (ALT), aspartate transaminase (AST), superoxide dismutase (SOD) and glutathione reductase (GR), glutathione peroxidase (GP), glutathione-S-tranferase (GST) and catalase (CAT) [123,125]. It should be pointed out that the normal cellular metabolism is the main source of endogenous ROS [140]. Accordingly, due to a lack of light-activation inside a digestive track, it should be assumed that an increase in ROS content is caused by a decrease in the level of enzymatic scavengers of antioxidant defense (in particular CAT, SOD, GST) and non-enzymatic scavengers (e.g., vitamin A, vitamin E, vitamin C). The overproduction of ROS could break the balance down between the oxidative and antioxidative systems, resulting in DNA damage and apoptosis. Besides, free radicals might exacerbate the lipid peroxidation, calculated on the basis of thiobarbituric acid reactive substances (TBARS) that are formed as by-products [125,130]. Fadda et al. [128] have indicated that after an oral exposure to titania an increase in production of inflammatory mediators (e.g., tumor necrosis factor-α, interleukin-6, C-reactive protein and immunoglobin G) and angiogenic factors (e.g., vascular endothelial growth factor VEGF) might induce both hepatic injury and distant organ damage.

Additionally, it should be pointed that consumption of titania has an impact on gut microbes. According to Pinget et al. [141] titania consumption from 2 to 50 mg TiO_2_/kg body weight per day does not influence significantly the microbiota composition in the small intestine and colon. In contrast, in vitro studies have shown the opposite results, i.e., titania impairs haemostasis and affects the spatial distribution of commensal bacteria. Moreover, it has been found that rutile decreases a number of probiotic genera, such as *Bifidobacterium sp*. and increases opportunistic pathogens genera, such as *Escherichia* sp. and *Shigella* sp. [138]. In animal studies, a potential autoimmune disease and cancer development have been also reported [133,138,142,143,144]. Additionally, it has been presented that titania NPs-injected mice, which possess implanted tumours, have exhibited the promotion of tumour metastasis and the presence of cancer cells in the blood vessels [145].

**Figure 7:**
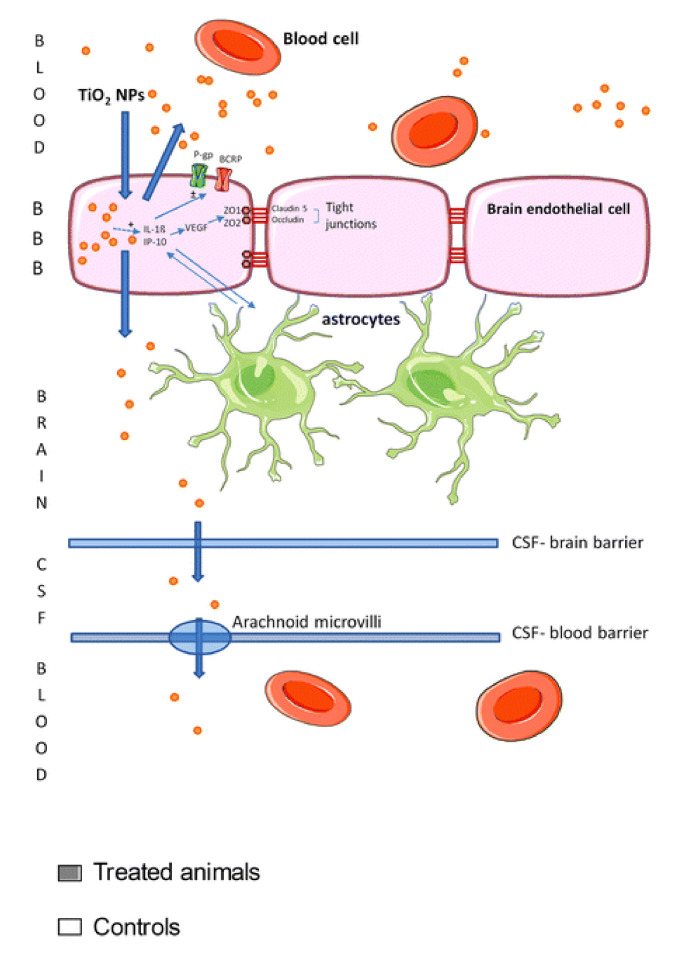
Schematic image of the events and potential effects on brain blood barrier (BBB) physiology when titania NPs are accumulated in organs. Adapted from reference [148].

In toxicity studies, the intraperitoneal injection (IP) involves the injection of probable toxin (here: titania suspension) into the peritoneum. The IP method has been used in few animal investigations [146,147,148,149,150]. Accordingly, it has been found that the injection of TiO_2_/NaCl suspension influences only slightly the rat lifespan, i.e., from 8% prolongation to 15% shortening [146]. However, the emotional behaviour of rats has been alerted, and the anxious index has increased [149]. For example, as shown by Disdier et al. [148] (Figure 7), the accumulated titania NPs in liver, lungs and spleen might be transported to the brain by blood, and thus titania-containing organs show an increase in the levels of tight junction proteins (claudin-5 and occludin), interleukin 1β (IL-1β), chemokine ligand 1 and γ inducible protein-10 in brain endothelial cells (which consists of the brain blood barrier (BBB)) and also IL-1β in the brain. One of the most important studies has been performed by Kreyling et al. [150] for quantitative analysis of NPs’ presence in all organs, tissues, carcass and excretion using radiolabeled [^48^V] TiO_2_ NPs. Consequently, it has been shown that regions of titania accumulation might be put in the following order: liver > spleen > carcass > skeleton and the blood. Interestingly, the titania content in the blood decreases rapidly after 24 h, whereas its distribution in other organs and tissues remains rather constant during the whole experiment [150]. Based on histopathological examinations, Chen et al. [147] have concluded that titania particles cause the lesion of the mouse spleen. Moreover, hepatocellular necrosis or apoptosis, hepatic fibrosis, renal glomerulus swelling, and interstitial pneumonia, associated with alveolar septal thickening, have also been noted. In contrast, completely different results have been found for rainbow trout (*Oncorhynchus mykiss*), where high doses of intravenously injected titania NPs (mixed-phase: rutile and anatase, with crystallite size of 23.2 nm) have caused a very limited (if any) overt impairment of renal function and an oxidative stress in the blood, despite the evidence of significant uptake and retention in those tissues [151].

### 3.3. Titania Ecotoxicity

It should be pointed out that broad application of titania NPs might have serious consequences for ecosystems. Titania as other oxides’ NPs might be spread in the environment by various ways, e.g., wastewater treatment streams, accidental release during production or daily applications (e.g., rinsing creams from the skin during swimming, paint leaching from an exterior facade of buildings/walls) [98,152]. It is expected that the behavior of titania NPs in the environment depends not only on its properties, but also on the features of the environment. Therefore, various titania–environment interactions, e.g., different environmental components (e.g., reducing/oxidizing agents, surfactants, organic matter and humid acids) and further titania transformations (e.g., aggregation/agglomeration, adsorption, deposition, dissolution, redox reactions, and interaction with macromolecules) are almost impossible to evaluate and predict reliably. Moreover, the bioavailability of titania depends also on its characteristics, environmental parameters and route of exposure [153]. For example, Kiser et al. [154] have shown that concentrations of titanium in effluents from wastewater treatment plants range from 0.005 to 0.015 g/dm^3^, whereas that accumulated in settled solids reaches concentrations from 1000 to 6000 µg/g. Therefore, it has been proposed that toxicity should be considered for various external conditions (background) before the assessment of the risk across the world [155]. The modelled data for titania NPs released into environmental compartments for different continents have revealed that in Europe the predicted environmental concentration of TiO_2_ in water, soil, sediments and effluents from sewage treatment plants is a few times higher than that in the U.S. [152]. Recently, it has been found that titania (similar to other chemical compounds, e.g., benzophenone-3 (BP-3)) tends to release from sunscreens into the sea and might be life-threatening for aquatic fauna and flora [156]. According to calculations made by the Labille et al., (2018) for one small beach visited by 3000 people daily, 68 kg of creams could be deposited per day, and thus 2.2 tons over the summer. Assuming that half of used creams contain 5% of titania, it should be expected that ca. 1.7 kg of titania might release to the sea per day, and ca. 54 kg during the two months of high summer [157]. It is well known that titania and other metal oxides’ NPs agglomerate, forming the micrometer particles, which might cause other hazardous effects to marine ecosystem (e.g., direct interactions with sedimented animals as annelids, worms and bivalves) [158,159]. However, nano-sized titania at a concentration of 10 mg/dm^3^ has shown much higher accumulation than bulk titania (> six fold higher), particularly in digestive gland of marine bivalves *Mytilus galloprovincialis* [160]. Additionally, NPs possess different dissolution/dispersion properties, depending on water properties (e.g., freshwater and seawater). For example, Canesei et al. [161] observed the formation of agglomerates of different sizes for different types of NP in artificial sea water. The most attention has been on photocatalytic reactions leading to ROS generation, when titania in seawater is exposed to UV radiation. Interestingly, it has been found that ROS, produced under these conditions, have a long lifetime and high steady-state concentrations [162].

Studies on the toxicity of sunscreens and personal care products on coral species started at the beginning of the 21^st^ century [163,164,165]. Many authors have argued that titania from sunscreens might be related to: (i) a decrease in biodiversity of marine ecosystems, (ii) worsening of the conditions of the coral reef, and (iii) affecting marine animals [158,159,160,162,166,167,168,169,170,171,172]. It has been found that under daylight illumination, nano-TiO_2_ is practically non-toxic for saline crustacea *Artemia salina*, and the EC_50(24h)_ and EC_50(48h)_ values have exceeded of 1.0 g/dm^3^ [172]. Nonetheless, an exposure to anatase and anatase/rutile nano-TiO_2_ under visible and UV light has increased the toxicity, showing values of EC_50(48h)_ from 4.03 to 4.05 mg/dm^3^ and from 284.81 to 408.67 mg/dm^3^, respectively [169]. Interestingly, much lower values, e.g., EC_50_ = 38.56 mg/dm^3^ for the dark exposure conditions and EC_50_ = 16.39 mg/dm^3^ for the light/dark exposure, have been presented for marine bivalve *Mytilus galloprovincialis* [159]. On the other hand, a short-term pilot study in situ conducted by the Coral Restoration Foundation has shown no toxicity when Caribbean scleractinian staghorn coral *Acropora cervicornis* has been exposed to 10 different brands of sunscreens [171].

**Figure 8:**
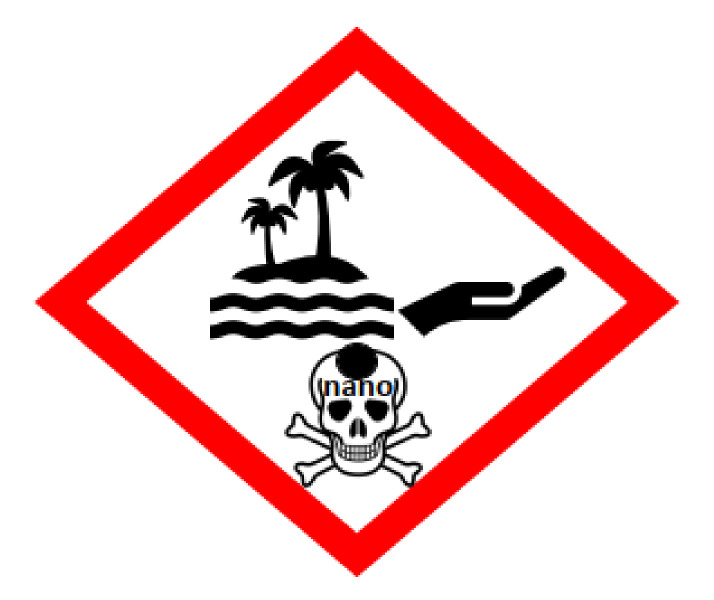
Proposed pictogram for commercial products containing inorganic NPs (e.g., titania), which might release when exposed to costal water.

It is worth noting that marine organisms are very often more sensitive to nano-TiO_2_ than freshwater ones. For example, the brine shrimp *A. salina* is more susceptible to titania than freshwater *Daphnia similis* [169]. The well-documented responses to NPs’ exposure include: (i) inhibition of growth rate, (ii) significant coral bleaching, (iii) change in feeding behaviors of marine animals, (iv) inhibition of larva development, and (v) increased pre-apoptotic processes in animal cells [159,164,166,168,169]. Galloway et al. [166] have shown that sublethal effects of titania to lugworm (*Arenicola marina*) correlate with damage by free radicals that can react with most DNA components (e.g., purine and pyrimidine bases and the deoxyribose backbone) and bind directly to DNA or DNA repair enzymes, leading to the formation of strand breaks. The genotoxicity of photoactivated titania at concentrations above 2 mg/g, and direct and indirect toxicities of nano-TiO_2_ aggregates at higher concentrations (>10 mg/dm^3^) have been shown for embryos of marine snail *Haliotis diversicolor* (by a coherent anti-stokes Raman scattering (CARS) microscopy technique) [158]. In the same study, it was also found that the toxicity is enhanced by tributyltin TBT—an antifouling compound, widely introduced into marine environments as antifouling paints. Because of that, it has been proposed that research on the impacts of titania NPs on corals should be carried out not only for sole NPs, but also for co-mixed components, e.g., titania with cosmetics and other chemical ingredients to simulate real commercial products, such as sunscreens and personal care products (PPCPs). Many sunscreen producers claim that their sunscreens contain UV filters, which are “reef safe”, but in fact, the majority of them have not been tested under in situ conditions. Unfortunately, there are no current regulations that enforce companies to undertake long-term toxicological studies in real conditions [173]. It is known that marine toxicology is challenging, and thus most aquatic toxicology studies are performed in the laboratory settings that do not mirror the environment, in which the organisms reside [171]. Moreover, there is a need to develop non-lethal monitoring methods, and improvements of universal practices and standards to determine the effects on various marine organisms. A comprehensive database to cumulate information on the effects on marine organisms and ecotoxicological endpoints should be also developed [174]. Additionally, it has been proposed that cosmetics and products consisting of titania NPs, e.g., sunscreens, could be labelled with the health hazards’ information for consumers, as well as pictograms (as proposed by us in Figure 8), presenting an environmental risk and precautionary statements for coastal waters [173].

Small planktonic invertebrates, such as *Daphnia magna* and *Thamnocephalus platyurus*, have been used in the majority of the studies for freshwater organisms [175,176]. Based on the lifecycle of crustaceans, the ecotoxicity chronic studies require a 21-day exposure period to establish survival and reproductive endpoints. Additionally, suspension-feeding organisms could incorporate NPs into their body very easily by endocytosis and phagocytosis. Unfortunately, the reported data are very confusing. For example, Heinlaan et al. [177] have shown that suspension of nano and bulk titania at a concentration of 20.0 g/dm^3^ are not toxic for *D. magna*, whereas Liu et al. [176] have estimated medial lethal concentration (LC_50_) of 0.5 g/dm^3^. Accordingly, Menard et al. [152] have proposed that the physicochemical properties of titania (crystal phases, specific surface area, average particle size) and the presence/absence of light (titania activation) determine the overall toxicity effect. The median effective concentration (EC_50_) for invertebrates (including *Mollusca*), defined as concentration of a substance in an environmental medium expected to produce a certain effect in 50% of test organisms, has been higher than 0.1 g TiO_2_/dm^3^ [152,161,169]. According to the results of acute toxicity experiments (short-term), titania NPs are non-lethal to daphnia, even up to EC_50_ concertation. The opposite results have been obtained from long-term studies, where titania at concentrations lower than 0.1 g/dm^3^ has shown toxic effects to crustacea, leading to increased mortality, growth decline and a lower number of offspring. Therefore, it is clear that exposure duration might be one of the most important factors of titania toxicity [178]. It has also been reported that 20 nm-sized TiO_2_ induces higher toxicity than 30 and 50 nm ones, because smaller NPs are more likely to enter inside the cells or/and accumulate under the carapace, as well as on the external body surface [176]. Moreover, the crystalline form of titania might be also important. For example, Clemente et al. [169] have shown that under the UV light exposure, the EC_50_ (48 h) for *Daphnia similis* is 12 times lower for anatase/rutile mixture than that for pure anatase.

One of the biggest problems is the transfer of titania particles in the trophic chain. Aquatic algae, phytoplankton and aquatic vascular plants, as well as higher plants in a non-aquatic environment, are the first trophic level in the food chain, and thus being a source for heterotrophic organisms. It has been proposed that sediments could be the main available sources of titania NPs in water. The model freshwater and marine algae, used in toxicology research, are microalgae species that have an ubiquitous distribution, e.g., *Pseudokirchneriella subcapitata*, *Desmodesmus subspicatusand*, *Chlamydomonas reinhardtii, Phaeodactylum tricornutum, Anabaena, Microcystis* and *Melosira* [152,170,179,180,181]. Once again, significantly variable values of median effective concentration EC_50_ for *P. subcapitata* have been reported for titania NPs, i.e., from 5.83 to 241.0 mg/dm^3^. Similar to invertebrates, the smaller titania particles (380 nm) are much more toxic than larger ones (>416 nm) also for algae. It is worth mentioning that tested species of microalgae are quite sensitive to the presence of toxic substances (including metals) and are broadly used in toxicology studies. Wang et al. [170] have reported that nano-TiO_2_ exerts most severe inhibition effects on *P. tricornutum* during the first day of exposure with a low EC_50_ value of 12.65mg/dm^3^. Additionally, more than 50% of the chlorophyll-a concentration in cyanobacteria (*Anabaena, Microcystis* and *Melosira*) has been reduced by TiO_2_-coated Pyrex hollow glass beads under illumination with UV-A light [179].

**Figure 9:**
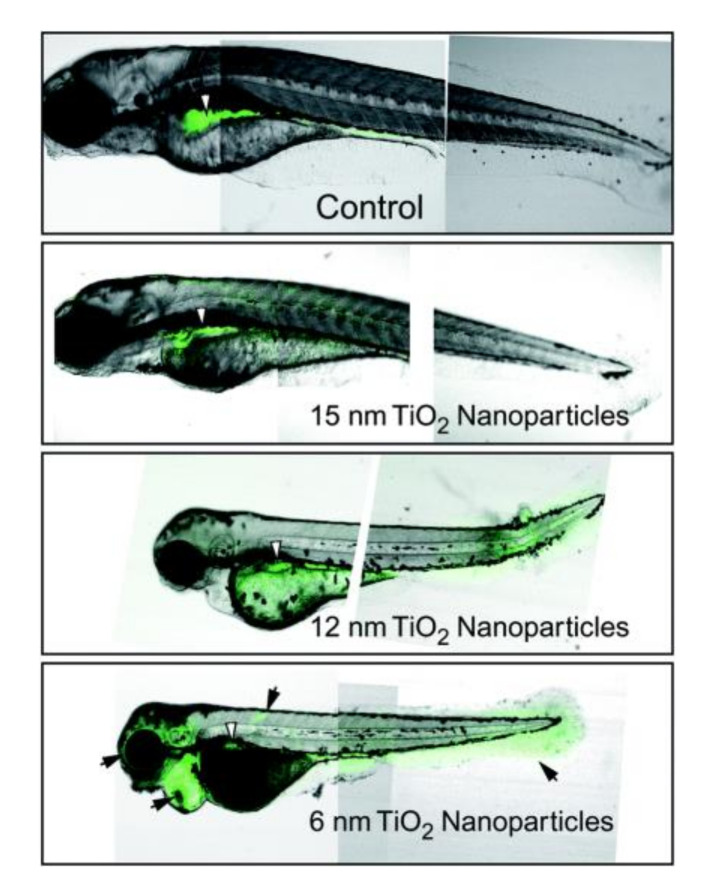
Fluorescence (reactive oxygen species (ROS) indicator, CMH2DCFDA staining) and bright field images of ROS generation in the zebrafish embryos after a treatment with titania NPs of different sizes, i.e., 6, 12 and 15 nm. White arrowheads, non-specific fluorescence. Black arrows, titania NP-specific signal. Adapted from reference [185] with permission from Royal Society of Chemistry, 2014.

Few works have been performed on titania toxicity towards higher water organisms, such as fishes, including Japanese rice fish or medaka (*Oryzias latipes*), zebrafish (*Danio rerio*) rainbow trout (*Oncorhyncus mykiss*) and carp (*Cyprinus carpio*) [151,176,182,183,184]. For example, ROS have been detected in zebrafish embryos in vivo after their treatment with titania NPs, and smaller titania NPs causes higher ROS generation (Figure 9) [185]. Moreover, Asztemborska et al. [186] have found that titania NPs could be transferred from *D. magna* to *D. rerio* by dietary exposure. The estimated LC_50_ of titania (particle size of 25 nm) for Japanese medaka is the same as that for *D. magna*, reaching 0.155 g/dm^3^. Moreover, it has been found that titania NPs have caused: reduced growth rate, decrease in the organ weight (e.g., liver), inhibition of antioxidant enzymes (e.g., CAT and SOD), destroyed gonadal tissue, reduced number of eggs, oxidative damage (e.g., lipid peroxidation) in cells and DNA, and histopathological changes of embryos and adults (e.g., thickening of edema and the gill lamellae) [151,176,183,187]. Some of these effects (e.g., pathological changes in the gill and liver) could cause fish suffering [188]. It has been shown that fish cells are generally more sensitive to oxidative injury than mammalian cells. Worrying toxicological data have been presented by Sun et al. [182] for nanocrystalline titania. It has been shown that nano-TiO_2_, due to its large specific surface area and the presence of surface hydroxyl groups, has a great adsorption capacity for highly toxic (to humans) arsenic ions (As(V)). The direct results of this adsorption might cause high accumulation of As/TiO_2_ in the stomach, intestine and gills of fishes. For example, after only 25 days of exposure to both titania and arsenate, arsenic concentration in carps has increased by 132% [182]). Moreover, arsenic might release from the titania surface, and then be uptaken by other organs, such as the liver and muscles. Similarly, it has been shown that titania NPs might increase an accumulation of other environmental toxins, e.g., cadmium (Cd) [189].

Agricultural contamination by titania is another aspect that should be considered. For example, contamination occurring in agricultural soil has caused an increase in Ti concentration up to 302% in sand. Fortunately, only low to negligible transfer to the soil leachate and the plant shoot has been noted [190]. Moreover, titania influences terrestrial ecosystems and causes changes in the microbial diversity and N cycle by decreasing of the denitrification directly and indirectly, and declining of the nitrification [191]. Drobne et al. [192] have found that the dietary exposure (3 and 14 days) of terrestrial isopod *Porcellio scabe* to titania NPs of 25 and 75 nm at concentrations of 10–1000 mg TiO_2_/g, i.e., much higher than that predicted for soil (0.0048 mg/g), enhances: (i) the feeding rate, (ii) the food absorption efficiency, and (iii) the activity of antioxidant enzymes CAT (catalase) and glutathione S-transferase (SOD). Fortunately, no adverse effects, such as mortality, body weight changes and reduced feeding, have been noticed. Quite opposite results have been found by Roh et al. [193] i.e., the toxic effect of 20-nm TiO_2_ particles to *Caenorhabditis elegans,* a free-living nematode [193]. Additionally, it has been confirmed that small NPs (7 nm) induce the ecotoxicity and affect: (i) *cyp35a* gene expression, (ii) fertility, and (iii) the survival of *C. elegants.* Hu et al. [194] have shown toxicological effects, induced by TiO_2_ NPs in the soil, on redworn *Eisenia fetida*. Moreover, it has been found that rutile (particle sizes of 10–20 nm) at concentrations above 1 g/kg bioaccumulates in the earthworms’ body and induces harmful effects, including oxidative stress, enzyme inhibition (cellulase), DNA damage and mitochondria damage [194].

**Figure 10:**
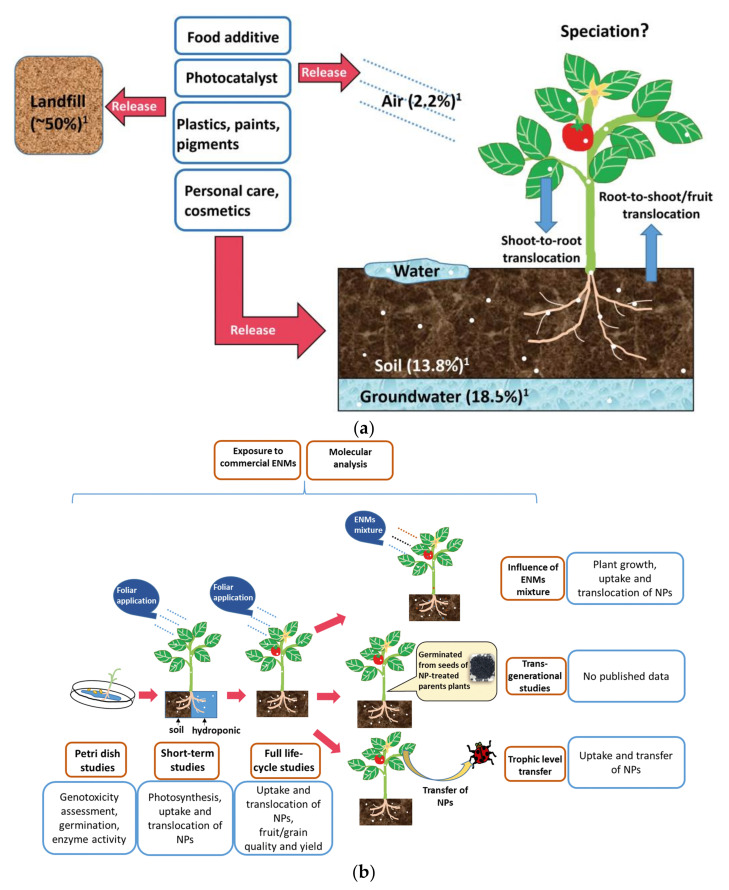
(**a**) Schematic representation of the titania interaction with plants; (**b**) trend of studies on the interaction between nano-TiO_2_ and plant systems. Blue box summarized the major findings in the study of each growth stage (orange box). Adapted from reference [197] with permission from Royal Society of Chemistry, 2018.

Although there are a limited number of papers concerning titania toxicity in higher plants [195,196]. Tan et al. [197] have summarized the interaction between nano-TiO_2_ and plants, including detection techniques, and possible effects of titania properties, such as the particle size, crystal phase, and surface coatings, as shown in Figure 10. Studies on the model plant *Aribidopsis thaliana*, crops and horticultural plants, such as *Allium cepa,* Avena sativa, *Cucumis sativus, Nicotiana tabacum, Spinacia oleracea, Zea mays, Oryza sativa, Brassica campestris ssp. napusvar*, Nippo-oleifera Makina, *Phaseolus vulgaris* var. humili, *Solanum lycopersicum Glycine max, Daucus carota* aquatic plants (e.g., *Lemna minor*), medical plants and willow tree *Salix* sp., have also been performed [152,195,196]. As summarized by Cox et al. [195], plants present various sensitivity to titania NPs. For example, for green algae *Desmodesmus subspicatus*, LC_50_ is 0.5g TiO_2_/dm^3^, whereas for the majority of other species, the value is larger than 1.0 g TiO_2_/dm^3^ [195,198]. Generally, plant basal metabolism and germination have not been affected by titania at a concentration of 2.0 g/dm^3^ [199]. It might be assumed that in some cases, titania might have even beneficial outcomes for some plants. For example, nano-anatase TiO_2_, which entered spinach cells easily, improves the whole chain of electron transport during photosynthesis, spinach growth and chlorophyll biosynthesis, and promotes the vigour of aged seeds [152,200]. Interestingly, improved root elongation and germination after exposition to titania at concentration of 20–40 mg/dm^3^ has also been found that could be associated with an increase in water uptake [54]. Similar results obtained by Andersen et al. [196] have shown that 1 g/dm^3^ titania (P25) does not cause widespread acute toxicity during germination and at early growth for 10 agronomic species. Although it has been known that NPs are uptaken by the seed coat, developing roots and leaves, the effects at later stages of the life cycle are still unknown. The toxic effects of small TiO_2_ particles (<100 nm) for *A. cepa* and *N. tabacum* have been caused by cellular oxidative stress, increased lipid peroxidation, antioxidative responses (enzyme activity) and induced DNA damage, e.g., chromosomal breaks, micronuclei formation, nuclear blebbing chromosome clumping and mitotic abnormalities [195].

Although an increase in ROS generation correlates well with TiO_2_-NPs uptake and titania properties (i.e., NPs’ size, surface morphology and physical properties), the toxicity effect does not depend on the exposition time. Interestingly, it has been shown that agglomeration of NPs (generation of larger particles than 100 nm) could be a limiting factor for the titania uptake by plants. Additionally, even after NPs’ uptake into plant metabolic systems, titanium does not exhibit a toxic potential [195]. Moreover, Klancnik et al. [201] have found that 15-nm TiO_2_ does not have genotoxic potential under dark conditions (without UV light).

**Figure 11:**
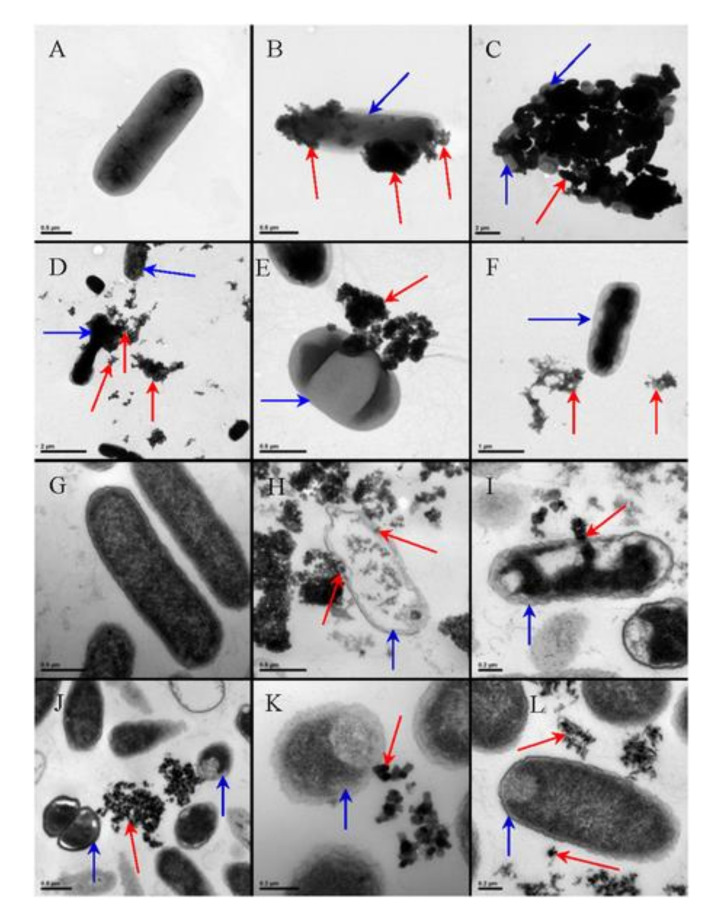
Transmission electron microscopy (TEM) images of *E. coli* bacteria unsliced (**A**–**F**) and sliced (**G**–**L**) without (**A**,**G**) and with the treatments of TiO_2_-NP 10A (**B**,**H**), TiO_2_-NP 25A (**C**,**I**), TiO_2_-NP 25AR (**D**,**J**), TiO_2_-NP 50A (**E**,**K**) and TiO_2_-NP 50R (**F**,**L**). Blue arrows, cells and red arrows, aggregated TiO_2_ NPs. Adapted from reference [216].

Titania NPs have been considered as safety antibacterial and antifungal agents for decades, as clearly reflected in a huge number of review papers concerning the application of titanium compounds as alternatives to antibiotic and antimicrobe agents [202,203,204,205,206,207,208,209,210,211]. This statement results from titania toxicity to prions, viruses, pathogenic Gram-positive and Gram-negative bacteria and microscopic fungi [212,213,214,215,216,217,218,219,220]. Most titania materials exhibit photocatalytic mechanism (by generation of ROS) of germ cells’ destruction. Titania likely interacts with cell walls and creates mechanical and chemical disruptions. Moreover, it has been found that the crystallite phase (anatase (A) or rutile (R) or mixture of them (AR)) and particle size influence the activity, as exemplary shown in Figure 11 [216].

**Figure 12:**
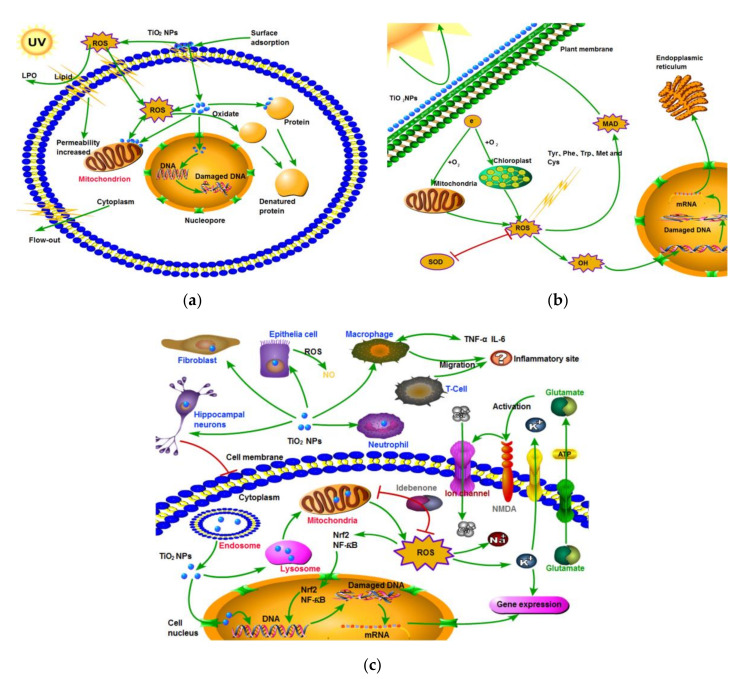
The mechanism of toxicity of titania NPs to: (**a**) microorganisms; (**b**) algae and plants; and (**c**) invertebrates and vertebrates. Adapted from reference [231] with permission from Elsevier, 2019.

Significant antibacterial activity of titania materials occurs only for suitable (determined experimentally for given conditions) concentrations of microorganisms, types of titania and methods of application (e.g., dissolution or immobilization). However, it has been reported that electrostatic interactions (determined by Zeta potential) between bacteria membranes and NPs are probably the most important for toxicity [216,221]. Additionally, such factors as nature and intensity of light illumination and characteristics of titania dopants/modifiers should be also considered [212,216,217,222,223]. It is thought that the form, size and morphology of particles are also very important factors [214,224,225,226,227,228,229]. In recent studies, Baysal et al. [230] have shown that the physicochemical characteristics of titania (e.g., agglomeration properties) are related to the environment type. Moreover, the content/composition of matrix influences titania’s fate and behavior in aquatic and soil environments. The mechanism of toxicity is similar to that described for other groups of organisms, including ROS interactions with cell-wall composites, lipid peroxidation of cell membranes, an inhibition of crucial antioxidant enzymes and DNA damage (Figure 12) [222,226,231,232]. However, the mechanism for different titania compositions has not been clarified yet [231,232].

## 4. Summary and Conclusions

The correct evaluation of the risk of titanium and its compounds requires understanding of all the factors involved in their behavior, as well as the generation of toxicity. As shown in this review, the effects of titanium and its compounds depend on the physicochemical properties, tested organisms, exposure methodology (e.g., in vivo or in vitro, ex situ or in situ), exposure time, illumination conditions, and thus full characterization of all factors must be considered when toxicity is discussed.

It might be concluded that there are no fully convincing studies proving that titanium implants and titania photocatalysts cause serious health and environmental problems. The potential carcinogenicity of titania powders to humans has been considered by authorities in some European Union (EU) countries and appropriate procedures are in progress. However, broad applications of titanium compounds result in their accumulation in various organisms and environments, thus disturbing environmental sustainability. Moreover, excessive accumulation of titanium (as well as any other element) poses a threat and thus cannot be ignored. Therefore, it should be carefully considered if the use of titanium and its compounds is necessary, reasonable, and causes more pros than cons.

Studies indicating titania toxicity (or the contact allergy) to human and animals cannot be ignored, and all people having direct contact with titania need to be aware of the sporadic problem of its noxiousness. Efforts should be made to obtain scientifically sound toxicity data from toxicity tests in the nearest future. It is believed that those data would result in avoidance of an unnecessary protection burden for industry (e.g., a need for personal protective equipment, PPE) and confusion for customers.

## Figures and Tables

**Table 1:** The reported cases of human health effects by titania.

Case	Contact	Exposed Persons	Results	References
1	13 years	53-year old man employed to pack titania	pneumoconiosis accompanied by right lung cancer	[100]
2	9 years	Man employed to pack titania into cans	slight fibrosis of interstitial lung tissue surrounding bronchioles and alveolar spaces after 5 years	[101]
3	-	55-year old man, heavily exposed to titania dust (rutile)	extensive pulmonary deposition of white pigment and absence of inflammatory and fibrotic changes	[101]
4	-	Four workers exposed to the inhalation of titania dust (not pure)	epithelioid granuloma, confirmed the inflammatory route of exposure	[102]
5	-	Four men and two women, between the ages of 22 and 65 years, unknown source of exposure	fibrosis and numerous macrophages with abundant deposition of a black pigment, confirmed presence of large quantity of titania in the pigment granule	[103]

## Abbreviations

ALT	Alanine aminotransferase
ANOVA	Analysis of variance
ANSES	French Agency for Food, Environmental and Occupational Health and Safety
AST	Aspartate transaminase
BBB	Brain Blood Barrier
BMSCs	Bone Marrow Stem Cells
BP-3	Benzophenone-3
CARS	Coherent Anti-stokes Raman Scattering and Coherent Anti-stokes Raman Scattering spectroscopy
CAT	Catalase
CLP	Classification, Labelling and Packaging
CMH2DCFDA	General Oxidative Stress Indicator, i.e., 5-(and-6)-chlomethyl-2′7′-dichlorodihydrofluorescein diacetate acetyl ester
DNA	Deoxyribonucleic acid
EC	European Council
EC50	Half maximal Effective Concentration
EU	European Union
ECHA	European Chemicals Agency
GP	Glutathione Peroxidase
GR	Glutathione Reductase
GST	Glutathione-S-Transferase
IARC	International Agency for Research on Cancer
IL	Interleukin
IP	Intraperitoneal injection
LD50	Median Lethal Dose
LDH	Lactate Dehydrogenase
MAK	Maximum Concentration values in the Workplace
MCF7	Breast cancer cells (Michigan Cancer Foundation-7 cell line)
MC3T3-E1	Mouse osteoblastic cells
mRNA	Messenger RNA
NF-κB	Nuclear Factor Kappa-light-chain-enhancer of activated B cells
NLM	National Library of Medicine
NIOSH	National Institute for Occupational Safety and Health
NOAEL	No-Observed-Adverse-Effect Level
NPs	Nanoparticles
OSHA	The Occupational Safety and Health Administration
P25	Evonik (Degussa) titanium dioxide (type P25)
PEL	Permissible Exposure Limit
PBS	Phosphate-Buffered Saline
PDL-hTERT	Periodontal Ligament human Telomerase Reverse Transcriptase
PPCPs	Pharmaceuticals and Personal Care Products
PPE	Personal Protective Equipment
RAC	Risk Assessment Committee
RANK-RANKL	Receptor Activator of Nuclear factor Kappa-Β–Receptor Activator of Nuclear factor Kappa-Β Ligand
RNA	Ribonucleic Acid
ROS	Reactive Oxygen Species
SOD	Superoxide Dismutase
TBARS	Thiobarbituric Acid Reactive Substances
TBT	Tributyltin
TDMA	Titanium Dioxide Manufacturers Association
TNF-α	Tumor Necrosis Factor α
TPS	Titanium Plasma-Sprayed
TWA	Time-Weighted Average
UV-A	Ultraviolet A
VEGF	Vascular Endothelial Growth Factor
YAP	Yes-Associated Protein
YNS	Yellow Nail Syndrome
